# Structural details of the enzymatic catalysis of carbonic anhydrase II via a mutation of valine to isoleucine

**DOI:** 10.1107/S2052252520014244

**Published:** 2020-10-30

**Authors:** Daumantas Matulis

**Affiliations:** a Department of Biothermodynamics and Drug Design, Institute of Biotechnology, Life Sciences Center, Vilnius University, Sauletekio 7, LT-10257 Vilnius, Lithuania

**Keywords:** carbonic anhydrase II, active-site mutation, enzymatic catalysis

## Abstract

Kim and co-workers [*IUCrJ* (2020). **7**, 985–994] advance our understanding of the catalytic mechanism of carbonic anhydrase II by studying a mutant V143I where the change (of one hydrophobic amino acid to another that differs by a single CH_2_ group) is probably the smallest alteration that can be introduced into a protein. The study was performed at high pressure in a CO_2_ atmosphere to visualize the bound substrate; it showed the behavior of the entrance conduit waters and the substrate alteration due to the mutation.

Carbonic anhydrase (CA) is one of the most studied proteins in molecular life sciences. It has been especially widely used as a biophysical model for protein stability and binding studies (Krishnamurthy *et al.*, 2008 ▸). CA is also one of the fastest known enzymes that catalyze the reversible hydration of carbon dioxide to bicarbonate anion and acid protons. Humans contain 15 highly homologous CA isoforms. Three of them do not perform the catalytic function of CA owing to the absence of Zn(II) in the active center as at least one of the three His residues holding the metal cation has changed to another amino acid. The remaining 12 isoforms have variable expression in tissues: some of them are localized intracellular in the cytosol or mitochondria, while others are located outside and attached to cell membrane via a lipid anchor or a transmembrane helix. Several of these isoforms have been implicated and are over-expressed in numerous cancers. Thus research in the CA field is quite active and has a significant impact on all sectors of protein science (Dodgson *et al.*, 1991 ▸; Chegwidden *et al.*, 2000 ▸; Frost & McKenna, 2014 ▸; Supuran & Simone, 2015 ▸; Matulis, 2019 ▸).

Since the discovery of sulfanilamide and acetazolamide in the 1940s, it has been established that primary sulfonamides are highly specific binders and inhibitors of CAs. Numerous sulfonamides bearing thia­zide, furosemide and other moieties have become clinically used drugs to treat hypertension and edema, as well as being used as diuretics. Dorzolamide and brinzolamide have been used as antiglaucoma agents. However, there is also a discussion on the applicability of some recently designed CA inhibitors (Jonsson & Liljas, 2020 ▸).

Since the first X-ray crystal structure of a CA (Liljas *et al.*, 1972 ▸), the atomic structures of ten human CA isoforms have been determined. The missing structures are isoforms expressed in mitochondria, CA VA and CA VB. However, isoform CA II is by far the most abundant in the PDB (Linkuvienė *et al.*, 2018 ▸). Most efforts to crystallize CA isoforms have been in the field of drug design for understanding how low molecular weight compounds recognize and distinguish CA isoforms. A good drug is supposed to selectively inhibit only one CA isoform, the target of a disease.

Despite these numerous studies, a truly detailed atomic level understanding of CA enzymatic activity, interaction of the enzyme with its substrate CO_2_ and especially the behavior of water molecules are not fully understood. The situation is further complicated by the substrate being gaseous making the methodology more complex.

Writing in this issue of **IUCrJ**, Kim *et al.* (2020 ▸) advance our understanding of the catalytic mechanism of CA II by making a mutation and comparing it with the native protein. The mutation (V143I) has already been previously introduced (Fierke *et al.*, 1991 ▸; West *et al.*, 2012 ▸). The change of valine to isoleucine is one of the smallest possible perturbations of a protein structure (where one hydro­phobic amino acid is changed to another hydro­phobic amino acid that differs by a single CH_2_ group) that can be introduced into a protein with the hope that such a change would cause almost no deviation in the catalytic properties of the enzyme.

The mutation actually changed the catalytic properties of the enzyme quite substantially. The *k*_cat_ value was not significantly affected, but the affinity of the substrate CO_2_ for CA II decreased tenfold, from 10 m*M K_m_* for CA II to 100 m*M K_m_* in the mutant. The study by Kim *et al.* (2020 ▸) demonstrates the structural arrangement of the CO_2_ in the active site and shows its diminished mobility in the mutant as a result of the decrease of available space in the substrate binding pocket.

As in a previous study where the pressurized CO_2_ was applied (Kim *et al.*, 2016 ▸), this study determined the crystal structures not only at ambient pressure in the air, but also at 7, 13 and 15 atmosphere pressure of CO_2_ gas (the 7 atm binding site is illustrated in Fig. 1 ▸). The concentration of CO_2_ in the experiment increased from approximately 300 p.p.m. in ambient air to more than 10 atm in the pressurized setting. Thus, the increase was approximately 30-thousand-fold, enabling visualization of the CO_2_ molecule bound in the active site of the protein. The mutation caused a shift of its position and partial conversion to an HCO_3_^−^ bicarbonate anion.

Furthermore, the study demonstrated that the proton transfer pathway around His64 has been largely unaffected, but there were subtle changes in the structure of the entrance conduit (EC) water molecules. It would be very interesting to see the positions of each proton in the conduit, but this would require neutron crystallography (Fisher *et al.*, 2012 ▸). Still, this remarkable study shows the effect of a mutation on the structure of the catalytic mechanism of CA II in the greatest detail so far and will be used in the design of compounds for pharmaceutical purposes.

## Figures and Tables

**Figure 1:**
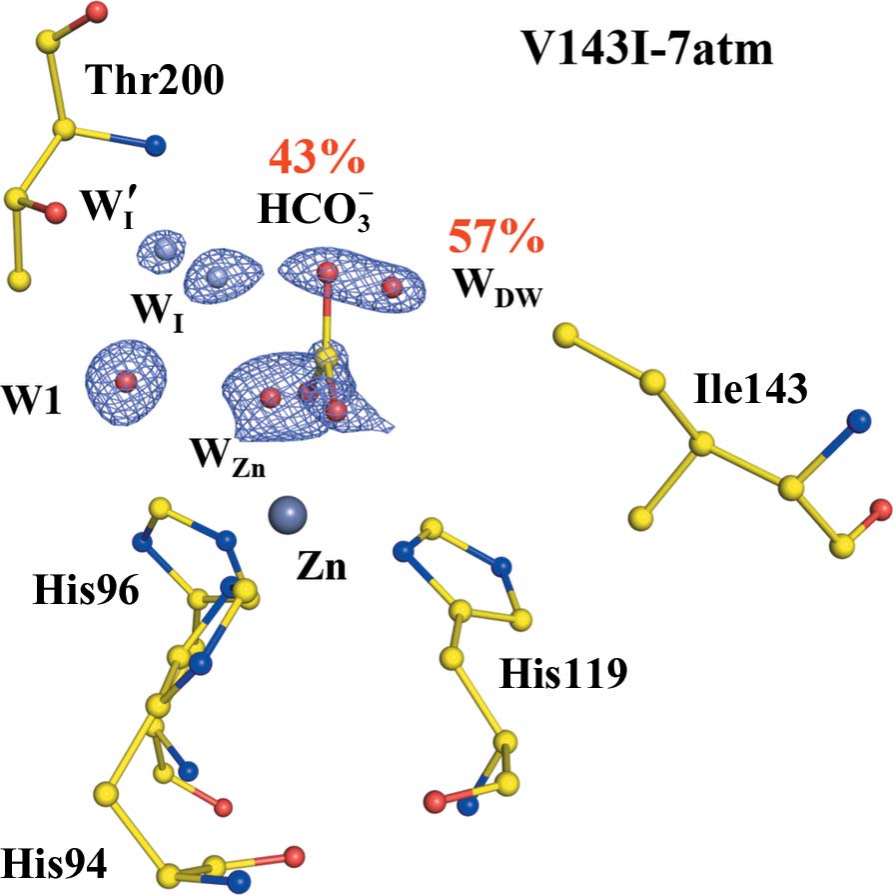
CO_2_/HCO_3_^−^-binding site of V143I CA II. The intermediate waters (W_I_ and W_I_′) are coloured steel blue for clarity. The electron density (2*F*_o_ − *F*_c_) is contoured at 1.5σ. Partial occupancies of HCO_3_^−^ and W_DW_ are shown. Reproduced from Kim *et al.* (2020 ▸).
